# Molecular Detection of Tick-Borne Pathogens in Kumasi: With a First Report of Zoonotic Pathogens in Abattoir Workers

**DOI:** 10.1155/2024/4848451

**Published:** 2024-07-14

**Authors:** Seth Offei Addo, Stacy Amoah, Nancy Martekai Unicorn, Emmanuella Tiwaa Kyeremateng, Genevieve Desewu, Patrick Kwasi Obuam, Richard Odoi-Teye Malm, Emmanuel Osei-Frempong, Francisca Adai Torto, Stephen Kwabena Accorlor, Philip Kweku Baidoo, Samuel K. Dadzie, John Asiedu Larbi

**Affiliations:** ^1^ Parasitology Department Noguchi Memorial Institute for Medical Research University of Ghana, Legon, Accra, Ghana; ^2^ Department of Theoretical and Applied Biology College of Science KNUST, Kumasi, Ghana; ^3^ School of Public Health Kwame Nkrumah University of Science and Technology, Kumasi, Ghana

**Keywords:** humans, livestock, tick-borne pathogens, zoonoses

## Abstract

Tick-borne pathogens continue to infect humans and animals worldwide. By adapting to the movement of livestock, ticks facilitate the spread of these infectious pathogens. Humans in close contact with animals that could be amplifying hosts are especially at risk of being infected with tick-borne pathogens. This study involved the collection of dry blood spots (DBSs) to determine tick-borne pathogens occurring in slaughtered livestock and abattoir workers in Kumasi. This study employed the use of conventional PCR, RT-PCR, and Sanger sequencing to detect and identify the tick-borne pathogens. The resulting data was analysed using Stata version 13. A total of 175 DBSs were collected from goats (76), cattle (54), and sheep (45) in the Kumasi abattoir (130, 74.29%) and Akwatia Line slaughter slab (45, 25.71%). The pathogens identified were mostly bacterial including *Anaplasma capra* (9.71%), *Anaplasma phagocytophilum* (1.14%), and *Rickettsia aeschlimannii* (0.57.%). The only parasite identified was *Theileria ovis* (9.14%). A significant association was seen between *A. capra* (*p* < 0.001) infection and female sheep sampled from the Akwatia Line slaughter slab. Again, there was a significant association between *T. ovis* (*p* < 0.001) infections and female sheep from the Kumasi abattoir. From the human DBS (63) screened, the pathogens identified were all bacterial including *Coxiella burnetii* (1.89%), *Rickettsia africae* (1.89%), and *R. aeschlimannii* (1.89%). This study reports the first detection of *R. aeschlimannii* in livestock as well as the occurrence of the above-mentioned pathogens in humans in Ghana. Animals can serve as amplifying hosts for infectious pathogens; hence, there is an increased risk of infections among the abattoir workers. Continuous surveillance effort is essential, and abattoir workers need to protect themselves from tick bites and infectious tick-borne pathogens.

## 1. Introduction

The importance of (re)emerging diseases that impact the health of humans and animals worldwide is increasing also due to tick's expansion and the pathogens that they carry [1, 2]. A study reports that diseases spread by ticks, including *A. phagocytophilum*, *Theileria*, and *Babesia*, represent a threat to more than 80% of cattle [3]. The global gross economic loss is between USD 13.9 and USD 18.7 billion a year [4].

In Africa, livestock are a valuable resource that improves owners' nutritional status and spurs economic development [5, 6]. However, these livestock can be hosts for infectious agents including tick-borne pathogens [7]. Due to the rapid population growth in Africa, there is a significant need for animal products, which has prompted cross-regional livestock trade and increased the danger of animal disease spread [8, 9]. To effectively utilize seasonal pasture resources, herders frequently move across international borders with their livestock [10]. Even though these activities may help infectious pathogens spread out, disease surveillance is often subpar or nonexistent close to the boundaries of many Sub-Saharan African nations [11]. As such, tick species and their associated pathogens can spread and invade new areas.

In the worldwide food supply chain, abattoirs play a crucial role and are widespread. Given that pre- and postslaughter supervision may not be rigorous, there is the worry that unsupervised abattoirs have a higher incidence of occupational health issues, including infections that spread from animals to people [12]. For personnel who come into contact with blood, uterine fluids, and placenta, infected livestock pose a risk. Pathogens can infect people at abattoirs, which can subsequently cause worker outbreaks locally [13] and spread throughout a population by ingestion and indirect or direct contact [14, 15]. Additionally, every carcass of an infected animal that is destroyed lowers the amount of food that can be produced for the community and lowers farmers' revenue.

In Ghana, imported livestock are brought in through the Burkina Faso border and down to Kumasi where the majority are slaughtered at the abattoir. Previous studies in Ghana have reported the seroprevalence of zoonotic pathogens in livestock from the Volta Region [16] and Kumasi [17, 18]. Furthermore, ticks infesting livestock have been reported to harbour zoonotic pathogens [17, 19, 20]. Although there is limited information on tick-borne pathogens in Kumasi, studies have reported the occurrence of *R. africae* and *R. aeschlimannii* in tick species [21] as well as *C. burnetii* exposure in sheep and goats [18]. This suggests a risk to abattoir workers and a need to adopt effective control measures. There are several benefits to using dry blood spots (DBSs) in resource-constrained settings for surveillance-based infectious disease diagnosis. These benefits include noninvasive blood collection, reduced blood volume needed, ease of transport, and long-term blood spot storage [22, 23]. Research has successfully identified tick-borne diseases in livestock DBSs [24–26]. This study sought to determine the tick-borne pathogens in slaughtered livestock and abattoir workers in Kumasi. The findings from this study will be useful in creating effective control measures to prevent zoonotic pathogens from spreading.

## 2. Methods

**Figure 1:**
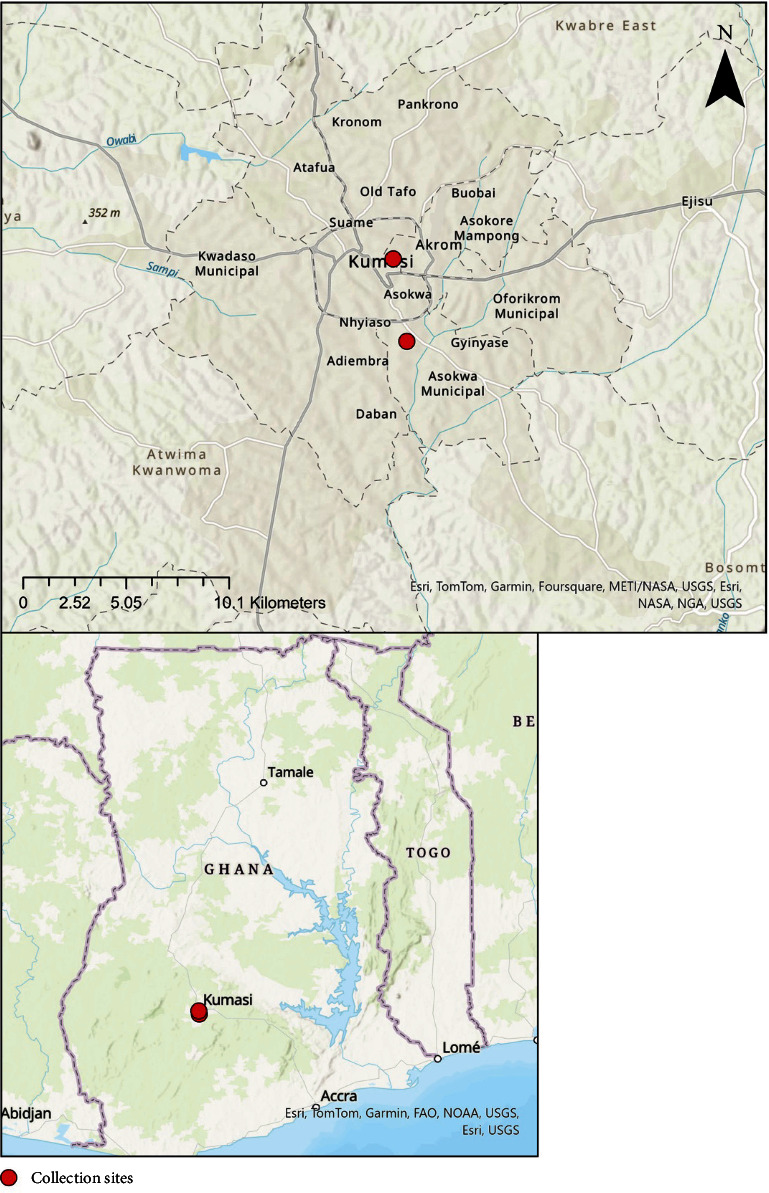
This map displays the specific locations where samples tested during this study were collected. It is important to note that the exact geographic origin of either animal or human hosts is unknown. Animals are often transported through Northern Ghana to Accra from multiple surrounding countries (e.g., Mali and Burkina Faso) while some animals are raised locally. It is not fully known how far slaughterhouse workers routinely travel from the sample collection sites. Future studies to fully characterize and map the movements of humans and animals are needed.

This study was conducted in the Kumasi abattoir and Akwatia Line slaughter slab where livestock are slaughtered to meet the demands of inhabitants in and around Kumasi (Figure 1).

Blood samples were collected from slaughtered livestock using Samco™ transfer pipettes (Thermo Scientific, United States) and spotted onto labelled FTA Gene cards (GE Whatman, Maidstone, Kent, United Kingdom), air-dried overnight, and stored in sample bags containing silica gel. Afterwards, the DBS samples were collected from the abattoir workers where the finger of each person was disinfected with an alcohol swab, pricked with a lancet, and spotted onto the FTA card. Each filter paper was then air-dried and stored in a labelled specimen bag containing silica gel. Using Epi info v5, a minimum of 141 livestock DBSs was required for this study.

The sample size was obtained based on the following assumption: a population size of 300 livestock, a prevalence rate of 22.4% [18], and a 95% confidence level with a 5% error margin. Furthermore, using Epi info v5, a minimum of 50 abattoir workers was required for the human DBS collection. The sample size was obtained based on the following assumption: a population size of 600 workers, a prevalence rate of 3.7% [18], and a 95% confidence level with a 5% error margin.

### 2.1. DNA Extraction and Molecular Detection of the Pathogens

From each DBS, DNA was extracted using Qiagen DNA Mini Kit (Qiagen Inc. Hilden, Germany) following the manufacturer's instructions and used as a template for pathogen screening. *Rickettsia* DNA in the DBS samples was detected using primers that target the rOmpA gene (ompA) of *Rickettsia*, amplifying a 632 bp fragment [27] (Table 1). Furthermore, *Ehrlichia* and *Anaplasma* DNA was identified in the DBS using primers that amplify a 345 bp fragment of the *Ehrlichia* genus 16SrRNA gene [30]. *Babesia*/*Theileria* DNA was also detected in the DBS using primers that amplify a 560 bp segment of the ssrRNA gene of *Babesia* and *Theileria* [29]. The above PCRs were performed in a Mastercycler X50-PCR Thermocycler (Eppendorf, Germany), with each reaction consisting of 5 *μ*L of DNA template, 1 *μ*M of each primer, 18 *μ*L of nuclease-free water, and 25 *μ*L of GoTaq® Hot Start Green Master Mix (2x). The resulting PCR products were separated on a 2% agarose gel, observed using a Molecular Imager® Gel Doc, and positive products were shipped to Macrogen Europe B.V. for purification and Sanger sequencing. *C. burnetii* DNA was detected in the DBS using a real-time PCR protocol that targets a 295-bp fragment of the transposase gene of the *C. burnetii* IS1111a element [28].

Using the Basic Local Alignment Search Tool, the sequences obtained in this investigation were compared to sequences in the NCBI database (BLAST). MEGA X was used to perform sequence alignments and phylogenetic analysis on the pathogens from this study [31]. The phylogenetic tree was built using the neighbor-joining approach. The confidence indices within the phylogenetic trees were calculated using 1000 bootstrap replicates, and results were displayed as percentages on the branches. The various accession numbers and nations of origin for the GenBank sequences used in the phylogenetic analysis have been listed.

### 2.2. Statistical Analysis

STATA version 13 was used to perform the statistical analysis. The chi-square test was used to determine the association between the occurrence of the identified pathogens and variables such as host, sex, and location. The significance level was set at *p* < 0.05.

## 3. Results

A total of 238 DBSs were collected from the Kumasi abattoir (183, 76.89%) and Akwatia Line slaughter slab (55, 23.11%). From the total DBS collected, 76 (31.93%) were from goats, 54 (22.69%) from cattle, 45 (18.91%) from sheep, and 63 (26.47%) from humans. The livestock sampled were composed of 105 (60%) females and 70 (40%) males compared to humans made up of 60 (95.24%) males and three (4.76%) females.

### 3.1. Pathogens Identified in the DBS

Pathogen DNA was detected in 32 (13.45%) of the sampled DBS with 12.18% occurring in livestock while 1.27% occurred in humans. Generally, with the livestock DBS, the pathogen DNAs identified were *A. capra* (7.14%), *A. phagocytophilum* (0.84%), *T. ovis* (6.72%), and *R. aeschlimannii* (0.42%). In the human DBS, the pathogen DNAs identified were *C. burnetii* (0.42%), *R. aeschlimannii* (0.42%), and *R. africae* (0.42%). All the infected humans were from the Kumasi abattoir (Table 2). Coinfections were seen in 2.94% of the livestock DBS, Kumasi abattoir (1.26%), and the Akwatia Line slaughter slab (1.68%). The occurring infections were *A. capra* and *T. ovis* in sheep. A significant association was seen between *A. capra* (*p* < 0.001) infection, sex, host, and location with most infections occurring in female sheep sampled from the Akwatia Line slaughter slab (Table 3). A significant association was also observed for *T. ovis* (*p* < 0.001) with most infections occurring in female sheep from the Kumasi abattoir.

*A. capra* in this study was 99%–100% similar to an isolate from Greece (ON415279) while *A. phagocytophilum* was 100% similar to an isolate from Denmark (AY776165). Again*, T. ovis* from this study was 99% similar to an isolate from Iraq (MN544931). While *R. africae* in this study was 99%–100% similar to an isolate from Benin (KT633262, KT633264), *R. aeschlimannii* was 95% and 100% similar to isolates from Lebanon (KY233239) and Turkey (MG920559), respectively.

**Figure 2:**
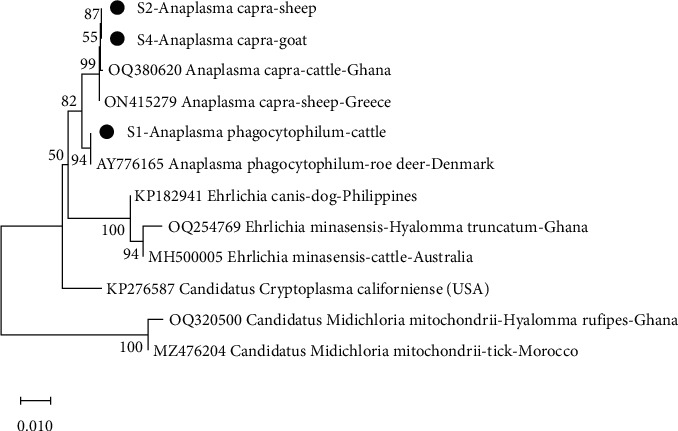
Phylogenetic analysis of *Anaplasma* based on the 16SrRNA gene. The sequences obtained in this study are indicated as S1, S2, and S4.

**Figure 3:**
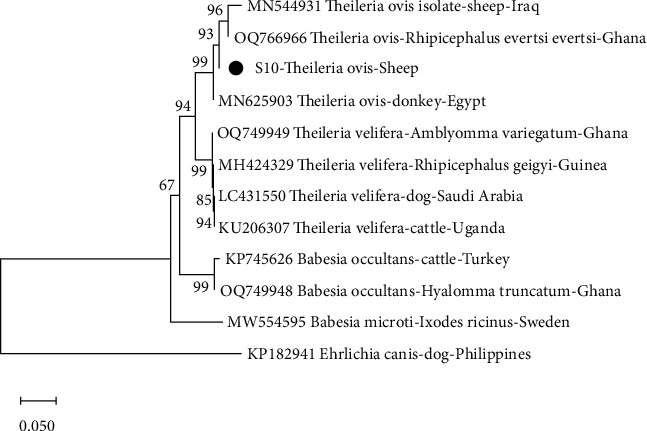
Phylogenetic analysis of *Theileri*a based on the ssrRNA gene. The sequence obtained in this study is indicated as S10.

**Figure 4:**
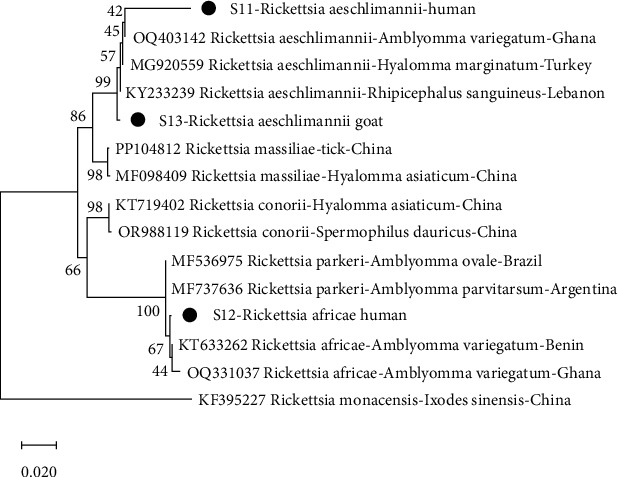
Phylogenetic analysis of *Rickettsia* species based on the OmpA gene. The sequences from this study are indicated as S11, S12, and S13.

From the phylogenetic analysis, *A. capra* from this study clustered with isolates from Ghana (OQ380620) and Greece (ON415279) with bootstrap support of 99% (Figure 2). With a 94% bootstra*p* value, *A. phagocytophilum* from this study also clustered with an isolate from Denmark (AY776165). *T. ovis* from this study clustered with isolates from Iraq (MN544931) and Ghana (OQ766966) with bootstrap support of 93% (Figure 3). It was observed that, with a bootstrap value of 67%, *R. africae* from this study clustered with isolates from Ghana (OQ331037) and Benin (KT633262) (Figure 4). Additionally, at 99% bootstrap value, *R. aeschlimannii* from this study clustered with isolates from Ghana (OQ403142), Turkey (MG920559), and Lebanon (KY233239).

The sequences generated in this study have been deposited in GenBank as follows: *A. phagocytophilum* (OR241137), *A. capra* (OR241135, OR241136), *T. ovis* (OR248172), *R. africae* (OR248872), and *R. aeschlimannii* (OR248871, OR248873).

## 4. Discussion

Previous studies in Kumasi have reported the tick species in the area to be mostly *A. variegatum*, *H. rufipes*, and *H. truncatum* with pathogen infection rates ranging from 0.3% to 14.29% [20, 21]. The pathogens reported were zoonotic including *R. africae*, *R. aeschlimannii*, and *C. burnetii*.

In this study, tick-borne pathogens of zoonotic and veterinary importance were identified in the sampled livestock and abattoir workers. Cattle in the Kumasi abattoir were found infected with *A. phagocytophilum* with no infections detected in cattle from the Akwatia Line slaughter slab. *A. phagocytophilum* is a zoonotic pathogen that infects humans and domestic and wild animals [32–34]. In cattle, infections result in fever, reduced milk production, and infertility [35]. The finding of this study can be compared to a previous study in Ghana that found *A. phagocytophilum* infection in cattle [36]. The first human case was reported in the United States [37] and has since spread to numerous countries including China [38] and Canada [39]. Infections can cause anything from a minor feverish sickness to serious multiple organ failure. Myalgia, headache, chills, and nonspecific fever are among the clinical signs and symptoms. The majority of infected humans only experience minor or no symptoms; however, serious systemic problems can happen, and about 36% of them need to be hospitalized [40]. It is important to note that there are different *A. phagocytophilum* strains with some being zoonotic while others are not [41].

To determine the strains circulating in Ghana, further studies will be essential. However, the detection of *A*. *phagocytophilum* DNA in cattle in the Kumasi abattoir necessitates further surveillance and management plans to stop its spread. Also in this study, *A. capra* DNA was identified in goats and sheep. This is a zoonotic pathogen that infects humans, ruminants, and wild animals [42–45]. Infections in goats can be mild or severe with symptoms such as weight loss, abortion, reduced milk production, and death [46, 47]. The only human cases of *A. capra* infections were reported in China [44] with the symptoms as fatigue, chills, headache, dizziness, and fever. However, another study has demonstrated that goat-derived *A. capra* infects human red blood cells [48]. This means that workers at the study sites in frequent contact with infected goats are at risk of infections. *A. capra* has been reported in ticks and livestock from the Upper East Region of Ghana [36]. Finding this pathogen in Kumasi could mean that the pathogen is distributed widely across the country through the trade and transport of livestock. Sheep in the sampled areas were also found infected with *T. ovis*. Small ruminants are often infected with *T. ovis* [49]. A recent study in Ghana has reported the occurrence of *T. ovis* in *R. evertsi evertsi* infesting sheep in the Upper East Region [50]. Infections with this pathogen are usually mild but can worsen when the animal is under stressful conditions or has a compromised immunity [51]. There is a need for more surveillance efforts in Ghana to determine the distribution of *T. ovis* and its effects on small ruminant production.

African tick-bite fever (ATBF), caused by *R. africae*, has gained relevance as the primary tick-borne rickettsiosis and the second most common cause of fever among travelers who visit Sub-Saharan Africa [52]. *R.* africae has been reported in a febrile patient from Zimbabwe following a tick bite [53]. Furthermore, *R. africae* has been reported in travelers who visited countries including South Africa [54–56], Namibia, Zimbabwe and Botswana [57], Ethiopia [58], Kenya [59], and Swaziland [60]. This study reports the first molecular detection of *R. africae* DNA in an abattoir worker in Kumasi, Ghana. With the high occurrence of *R. africae* in *A. variegatum* in Ghana [61], there is an increased risk of infections to the abattoir workers who are in direct contact with livestock that may be infested with *R. africae-*infected ticks. Some symptoms in infected individuals include headache, chills, fatigue, myalgia, and malaise [62]. The first documented human case of *R. aeschlimannii* infection was reported in a patient from France who had visited Morocco [63]. Again, *R. aeschlimannii* was detected in a patient in South Africa upon returning from a fishing and hunting trip [64]. In Tunisia, patients suspected of clinical rickettsial infection were found exposed to *R. aeschlimannii* [65]. *R. aeschlimannii* infection has also been reported in humans from Algeria [66] and Greece [67]. This study reports the first molecular detection of *R. aeschlimannii* DNA in a male worker as well as a goat in the Kumasi abattoir. It is plausible that the worker got exposed to the pathogen in the process of handling infected livestock or through a tick bite. Animals can serve as amplifying hosts for infectious pathogens; hence, there is an increased risk of infections among the abattoir workers [7].

This study also reports the first molecular detection of *C. burnetii* DNA in an abattoir worker in Ghana. *C. burnetii* infects humans when they come into contact with infected domestic animals such as cattle, sheep, goats, and dogs [68]. *C. burnetii* exposure in sheep, cattle, and goats has been reported from the Volta Region of Ghana [16]. More recently, a study in the Kumasi abattoir indicated the seroprevalence of *C. burnetii* in slaughtered goats and sheep [18]. Finding *C. burnetii* DNA in an abattoir worker in the Kumasi abattoir suggests that the workers are at risk of infections due to their frequent contact with livestock. Infected livestock can transmit *C. burnetii* through their milk, faeces, and most importantly, birth fluids and placental tissues [69, 70]. The main method of infection in humans is through inhalation of infected aerosols; however, alternative methods of pathogen transmission include person-to-person transfer, skin contact, and intake of dairy products [71]. In some situations, the human *C. burnetii* infection advances to a chronic stage characterized mostly by vascular infection or endocarditis, even though it usually remains undetected or manifests as a flu-like sickness, hepatitis, or pneumonia [72, 73].

Abattoir workers need to be educated on the threats posed by tick-borne pathogens. There is a need to ensure that each worker uses gloves and protective gowns when handling the livestock or their body parts. The workers should also be encouraged to regularly check themselves for ticks and actively avoid tick bites.

## 5. Conclusion

This study reports the first molecular identification of zoonotic pathogens *R. africae*, *R. aeschlimannii*, and *C. burnetii* in abattoir workers in Ghana. Furthermore, the livestock sampled were found to harbour *A. capra* and *A. phagocytophilum* which are of zoonotic and veterinary importance. The findings suggest an increased risk of pathogen transmission to the abattoir workers and a need to adopt effective control and preventive measures.

## Figures and Tables

**Table 1:** Primers and probe used in this study.

**Pathogen DNA**	**Target**	**Name**	**Sequence**	**Annealing temperature (°C)**	**References**
			*RT-PCR*		
*Coxiella burnetii*	*C. burnetii* IS1111a element	Cox-F	CCCCGAATCTCATTGATCAGC	60	Klee et al. [28]
		Cox-R	CCCCGAATCTCATTGATCAGC		
		Cox-TM	FAM-AGCGAACCATTGGTATCGGACGTT-TAMRA-TATGG		
			*Conventional PCR*		
*Rickettsia* spp.	rOmpA gene	190-70F	ATGGCGAATATTTCTCCAAAA	51.5	Jiang et al. [27]
		190-701R	GTTCCGTTAATGGCAGCATCT		
*Babesia*/*Theileria*	ssrRNA gene	Forward	GTCTTGTAATTGGAATGATGG	50	Beck et al. [29]
		Reverse	CCAAAGACTTTGATTTCTCTC		
*Ehrlichia*/*Anaplasma*	16SrRNA gene	16SF	GGTACCYACAGAAGAAGTCC	52.8	Nazari et al. [30]
		16SR	TAGCACTCATCGTTTACAGC		

**Table 2:** Distribution of tick-borne pathogens in the livestock and humans.

**Host**	**Sampling site**	**Total no. of DBS**	**Pathogens detected**	**No. of positive (%)**
Goat	Kumasi abattoir	60	*A. capra*	2 (3.33)
			*R. aeschlimannii*	1 (1.67)
	Akwatia Line	16	*A. capra*	3 (18.75)
Cattle	Kumasi abattoir	51	*A. phagocytophilum*	2 (3.92)
	Akwatia Line	3	None	
Sheep	Kumasi abattoir	19	*A. capra*	3 (15.79)
			*T. ovis*	11 (57.89)
	Akwatia Line	26	*A. capra*	9 (34.62)
			*T. ovis*	5 (19.23)
Human	Kumasi abattoir	53	*C. burnetii*	1 (1.89%)
			*R. aeschlimannii*	1 (1.89%)
			*R. africae*	1 (1.89%)
	Akwatia Line	10	None	

**Table 3:** Association between the identified pathogens, livestock, human, location, and sex.

**Factors**	**No. of DBS**	***A. capra***		***A. phagocytophilum***		***T. ovis***		***C. burnetii***		***R. africae***		***R. aeschlimannii***	
		**No. of positive**	**p** **value**	**No. of positive**	**p** **value**	**No. of positive**	**p** **value**	**No. of positive**	**p** **value**	**No. of positive**	**p** **value**	**No. of positive**	**p** **value**
*Host*													
Cattle	54	0	< 0.001	2	0.076	0	< 0.001	0	0.425	0	0.425	0	0.69
Goat	76	5		0		0		0		0		1	
Sheep	45	12		0		16		0		0		0	
Human	63	0		0		0		1		1		1	
*Location*													
Akwatia Line	55	12	< 0.001	0	0.463	5	< 0.001	0	0.583	0	0.583	0	0.463
Kumasi abattoir	183	5		2		11		1		1		2	
*Sex*													
Female	108	16	< 0.001	1	0.895	16	< 0.001	0	0.361	0	0.361	0	0.196
Male	130	1		1		0		1		1		2	

## Data Availability

All the data have been included in this article.
